# A rare der(10)t(X;10)(p11.2;p11.2) in an elderly patient with therapy‐related acute myelomonocytic leukemia

**DOI:** 10.1002/jha2.360

**Published:** 2021-12-01

**Authors:** Masahiro Manabe, Nao Tanizawa, Satoru Nanno, Yuuji Hagiwara, Reiko Asada, Ki‐Ryang Koh

**Affiliations:** ^1^ Department of Hematology Osaka General Hospital of West Japan Railway Company Osaka Japan; ^2^ Department of Clinical Laboratory Osaka General Hospital of West Japan Railway Company Osaka Japan

An 80‐year‐old male was referred to our hospital with dyspnea on effort. He had a history of diffuse large B‐cell lymphoma 2 years ago, for which he was treated with chemotherapy, including rituximab, cyclophosphamide, vincristine, doxorubicin, and prednisolone. His laboratory data included a white blood cell count of 1.9 × 10^9^/L, a hemoglobin concentration of 10.5 g/dl, and a platelet count of 92 × 10^9^/L. His serum lactate dehydrogenase level was 196 U/L. A bone marrow examination revealed a hypercellular bone marrow with a blast frequency of 23.4%. The blast cells were positive for both nonspecific esterase and naphthol AS‐D chloroacetate esterase staining (Figure 1). A cytogenetic study of the patient's bone marrow cells demonstrated the following karyotype: 47,XY, +8, der(10)t(X;10)(p11.2;p11.2)[7]/46,XY[13] (Figure 2). A diagnosis of therapy‐related acute myelomonocytic leukemia was made due to his history of chemotherapy‐treated lymphoma. Although the patient received two courses of azacitidine monotherapy, it was not effective, and he died 3 months after being diagnosed with leukemia.

**FIGURE 1:**
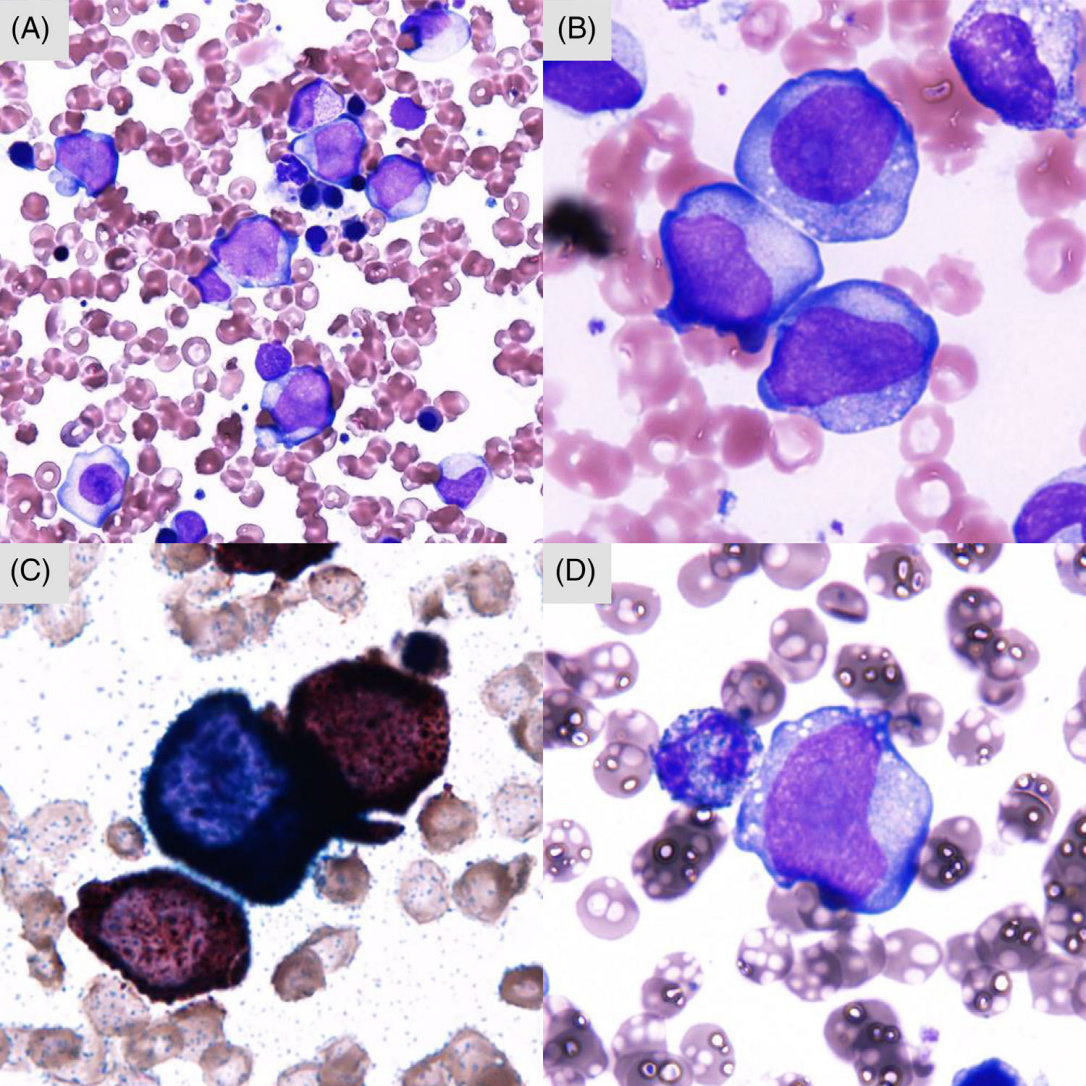
Bone marrow cells seen at the diagnosis of leukemia. (A and B) May‐Giemsa‐stained blasts are shown. The blasts were positive for esterase staining (C) and negative for myeloperoxidase staining (D). (magnification: A: 400×; B–D: 1000×)

**FIGURE 2:**
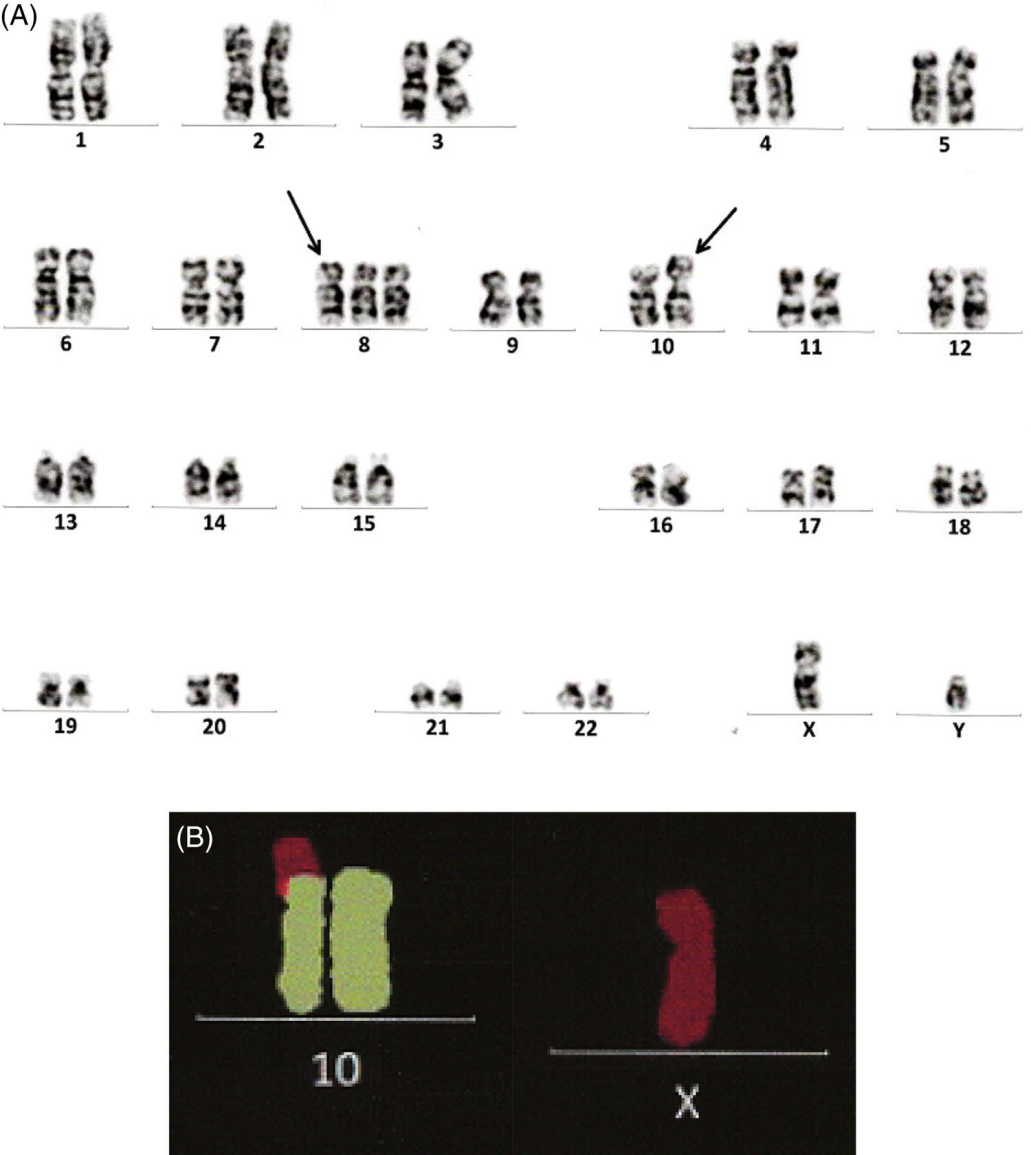
(A) The G‐banded karyogram obtained in this case; (B) partial multicolor fluorescent in situ hybridization (mFISH) analysis

To the best of our knowledge, only five cases of acute myeloid leukemia (AML) involving a translocation consisting of 10p11 and Xp11 breakpoints have been reported [1, 2, 3, 4, 5]. Of these, one case demonstrated the t(X;10)(p11;p11) translocation as a sole abnormality [3]. This translocation has never been reported in other solid tumors. Hence, t(X;10)(p11;p11) is considered to be a rare, but recurrent, cytogenetic aberration in AML. The reported cases have several things in common, that is, they all involved balanced translocations, most of them involved therapy‐related leukemia, and all of the patients except one (a 50‐year‐old [5]) were children. Although our case also involved therapy‐related leukemia, it was very rare, as it involved an 80‐year‐old patient who harbored an unbalanced der(10)t(X;10)(p11;p11) translocation.

As for the association between t(X;10)(p11;p11) and the pathogenesis of AML, our case involved der(10)t(X;10)(p11;p11), but lacked der(X)t(X;10)(p11;p11) due to the unbalanced nature of the translocation. Therefore, der(10)t(X;10)(p11;p11), rather than der(X)t(X;10)(p11;p11), is proposed to play a crucial role in the leukemogenesis in t(X;10)(p11;p11)‐harboring AML.

## INFORMED CONSENT

The patient's consent was obtained for the publication of the case report.

## CONFLICT OF INTEREST

The authors declare no conflict of interest.

## AUTHOR CONTRIBUTIONS

Masahiro Manabe, Nao Tanizawa, Satoru Nannno, and Ki‐Ryang Koh designed the study. Masahiro Manabe, Yuuji Hagiwara, and Reiko Asada analyzed the data. Masahiro Manabe and Ki‐Ryang Koh collected the clinical data and specimens. Masahiro Manabe, Yuuji Hagiwara, and Reiko Asada wrote the manuscript. All of the authors have reviewed and approved the final manuscript.

## Data Availability

All data generated during this study are included in this manuscript.
